# Serum vitamin D levels correlate with metabolic abnormalities and microalbuminuria in diabetic patients: a restricted cubic spline dose–response analysis

**DOI:** 10.3389/fnut.2026.1811665

**Published:** 2026-05-19

**Authors:** Yanmei Lin, Jianqing Tian, Kang Du, Yingji Wang

**Affiliations:** 1 Fujian Medical University Xiamen Humanity Hospital, Xiamen City, Fujian, China; 2 Zoe Soft Co. Ltd., Xiamen, Fujian, China; 3 Department of Geriatric Medical Center, Sichuan Provincial People’s Hospital, University of Electronic Science and Technology of China, Chengdu, China

**Keywords:** ACR, diabetes mellitus, metabolic parameters, microalbuminuria, vitamin D

## Abstract

**Objective:**

This study aimed to explore the dose–response associations of serum vitamin D levels with metabolic parameters and microalbuminuria (ACR) in diabetic patients, to identify the critical clinical threshold of vitamin D for renal and metabolic benefits, and to clarify the population heterogeneity of these associations stratified by microalbuminuria status.

**Methods:**

A total of 485 diabetic patients were retrospectively enrolled and divided into four groups according to vitamin D quartiles: Q1 (≤32.37 nmol/L, *n* = 121), Q2 (32.38–45.70 nmol/L, *n* = 121), Q3 (45.71–59.98 nmol/L, *n* = 122), and Q4 (≥59.99 nmol/L, *n* = 121). Spearman’s correlation analysis, multivariate linear regression analysis, and restricted cubic spline (RCS) modeling were used for statistical analyses, with a stratified analysis performed by microalbuminuria status.

**Results:**

The median serum vitamin D level was 45.7 nmol/L in this cohort, with a vitamin D deficiency/insufficiency rate of 75.1%. Serum vitamin D levels were significantly inversely correlated with HbA1c (*ρ* = −0.425), fasting glucose (*ρ* = −0.386), triglycerides (*ρ* = −0.358), BMI (ρ = −0.302), and ACR (ρ = −0.286) (all *p* < 0.001). Compared with Q4, Q1 patients had a 1.95% higher HbA1c (95% CI: 1.52–2.38) and a 68.95 mg/g higher ACR (95% CI: 50.12–87.78). RCS analysis revealed a linear dose–response relationship between vitamin D (20–50 nmol/L) and reductions in HbA1c (0.32% per 10 nmol/L) and ACR (12.75 mg/g per 10 nmol/L), with a plateau effect observed above 50 nmol/L. The inverse association between vitamin D and ACR was significantly stronger in patients with microalbuminuria (*β* = 75.68 vs. 43.52, interaction *p* < 0.001).

**Conclusion:**

Vitamin D levels are negatively associated with metabolic abnormalities and microalbuminuria in a dose-dependent manner, suggesting that patients with microalbuminuria may have a stronger association, warranting further investigation in prospective studies.

## 1 Introduction

Diabetes mellitus (DM) has become a major global public health challenge, with an estimated prevalence of 11.2% among Chinese adults (1). As a chronic metabolic disorder, DM is often accompanied by multiple metabolic abnormalities (e.g., glycemic disorders, dyslipidemia, and obesity) and is closely associated with the development of various complications (2). Diabetic kidney disease (DKD) is one of the most common and severe microvascular complications of DM, affecting approximately 30–40% of diabetic patients (3). Microalbuminuria, defined as a urinary albumin-to-creatinine ratio (ACR) of 30–300 mg/g, is an early and critical marker of renal injury in DM, and its occurrence significantly increases the risk of progression to end-stage renal disease and cardiovascular events (4). Therefore, identifying modifiable risk factors for metabolic abnormalities and early renal injury in diabetic patients is crucial for improving their long-term prognosis.

Vitamin D, traditionally recognized for its role in regulating calcium-phosphorus metabolism and bone health, has recently been found to exert extensive physiological effects beyond bone metabolism (5). Vitamin D receptors are widely expressed in pancreatic *β*-cells, adipocytes, hepatocytes, and renal tissues, participating in the regulation of insulin secretion, lipid synthesis, energy metabolism, and renal function (6, 7). Accumulating epidemiological evidence suggests that vitamin D deficiency is highly prevalent in diabetic patients and may be associated with poor metabolic control and an increased risk of DKD (8, 9). However, existing studies on the associations between vitamin D and metabolic parameters or microalbuminuria in diabetic patients have yielded inconsistent results. Some studies have reported a negative correlation between vitamin D levels and glycated hemoglobin (HbA1c) or triglycerides (TG), while others have failed to confirm such associations (10, 11). Moreover, few studies have explored the dose–response relationship between vitamin D and these outcomes, and the potential heterogeneity of associations across different populations (e.g., patients with or without microalbuminuria) remains unclear (12).

In this context, the present retrospective cross-sectional study was conducted on 485 diabetic inpatients. We aimed to (1) investigate the prevalence of vitamin D deficiency/insufficiency in diabetic patients; (2) explore the associations between serum vitamin D levels and metabolic parameters (HbA1c, fasting plasma glucose [FPG], low-density lipoprotein cholesterol [LDL-C], TG, and BMI) as well as microalbuminuria (ACR); (3) clarify the dose–response relationship between vitamin D and key outcomes using restricted cubic spline (RCS) models; and (4) assess the heterogeneity of associations by stratifying patients according to the presence of microalbuminuria. The findings of this study may provide clinical evidence suggesting associations between vitamin D status and metabolic/renal parameters in diabetic patients, which could inform future interventional studies on the comprehensive management of diabetes and its complications (13, 14).

## 2 Subjects and methods

### 2.1 Subjects

A total of 485 diabetic inpatients admitted to the Department of Endocrinology of our hospital from January 2024 to June 2025 were retrospectively enrolled. The diagnosis of diabetes mellitus was strictly in accordance with the criteria specified in the Guidelines for the Prevention and Treatment of Type 2 Diabetes Mellitus in China (2022 edition). The inclusion criteria were as follows: (1) age ≥ 18 years old and (2) complete clinical medical records, with all patients undergoing measurements of serum vitamin D levels, glycated hemoglobin (HbA1c), fasting plasma glucose (FPG), blood lipids, body mass index (BMI), and urinary albumin-to-creatinine ratio (ACR). (3) This retrospective study was approved by the Ethics Committee of Fujian Medical University Xiamen Humanity Hospital. Due to the retrospective design and use of anonymized data, the requirement for written informed consent was waived. The exclusion criteria were as follows: (1) hepatic or renal insufficiency (liver function indicators exceeding two times the upper limit of normal, estimated glomerular filtration rate (eGFR) < 60 mL/min·1.73m^2^); (2) presence of malignant tumors or autoimmune diseases; (3) administration of vitamin D supplements, glucocorticoids, or other drugs that may affect metabolism or renal function within the past 3 months; (4) pregnant or lactating women; (5) and massive proteinuria (ACR ≥ 300 mg/g) or end-stage renal disease patients.

### 2.2 Definition and grouping of variables

#### 2.2.1 Exposure variable

Serum 25-hydroxyvitamin D [25(OH)D] level (nmol/L) was measured by electrochemiluminescence immunoassay (Elecsys Vitamin D Total, Roche Diagnostics, Mannheim, Germany). For descriptive purposes, patients were categorized according to the 2022 Guidelines for the Diagnosis and Treatment of Vitamin D Deficiency and Insufficiency into three groups: vitamin D deficiency (<25 nmol/L), insufficiency (25–50 nmol/L), and sufficiency (>50 nmol/L). They were also divided into four groups according to vitamin D quartiles: Q1 (≤32.37 nmol/L, *n* = 121), Q2 (32.38–45.70 nmol/L, *n* = 121), Q3 (45.71–59.98 nmol/L, *n* = 122), and Q4 (≥59.99 nmol/L, *n* = 121).

#### 2.2.2 Outcome variables

(1) The metabolism-related indicators were as follows: HbA1c (%), FPG (mmol/L), low-density lipoprotein cholesterol (LDL-C, mmol/L), triglycerides (TG, mmol/L), and BMI [kg/m^2^, calculated as body weight (kg) divided by the square of height (m)]; (2) The renal-related indicator was as follows: ACR (mg/g), with microalbuminuria defined as an ACR 30–300 mg/g.

Confounding variables included age (years), sex (male/female), smoking status (yes/no, defined as cumulative smoking of ≥ 1 year and ≥ 1 cigarette per day), hypertension (yes/no, defined as systolic blood pressure ≥ 140 mmHg and/or diastolic blood pressure ≥ 90 mmHg, or previously confirmed hypertension with regular medication), and duration of diabetes (years, defined as time from diabetes diagnosis to this hospitalization).

### 2.3 Statistical analysis

SPSS 26.0 and R version 4.2.0 software (rms and ggplot2 packages) were used for statistical analysis, with a two-tailed *p*-value of < 0.05 considered statistically significant. Continuous variables were non-normally distributed, as tested by the Shapiro–Wilk test, and were expressed as medians (interquartile ranges) [M (Q1, Q3)] or mean ± standard deviation (x ± s); intergroup comparisons were performed using the Kruskal–Wallis H test or a one-way analysis of variance (ANOVA). Categorical variables were expressed as frequencies (percentages) [*n* (%)], and intergroup comparisons were conducted using the chi-squared test. Spearman’s correlation analysis was used to assess the correlation between serum vitamin D levels and each outcome variable. A multivariate linear regression model was constructed to analyze the independent effect of vitamin D grouping on metabolic indicators and ACR after adjusting for age, sex, smoking status, hypertension, and duration of diabetes. Restricted cubic spline (RCS) modeling (with 3 knots at P25, P50, and P75) was used to explore the dose–response relationship between vitamin D, HbA1c, and ACR. Stratified analysis was performed according to the presence or absence of microalbuminuria, and interaction terms were introduced to test for heterogeneity of the associations across subgroups. Dose–response curves, forest plots, and boxplots were drawn using the ggplot2 package, and graph production complied with medical journal standards. To formally test for a potential threshold effect in the dose–response relationships, piecewise linear regression was performed using the ‘segmented’ package in R (version 4.2.0). The breakpoint (threshold) was estimated using a grid search approach based on the maximum likelihood method. The statistical significance of the slope change (i.e., the difference in slopes before and after the breakpoint) was assessed using the Davies test. The analysis was adjusted for the same set of covariates as in the main multivariate linear regression models.

To assess the robustness of the multivariate regression models, we performed several diagnostic analyses. Multicollinearity was assessed using variance inflation factors (VIFs), with VIF values < 5 indicating no significant multicollinearity. Model assumptions were evaluated by examining residual plots for homoscedasticity and normality (Q-Q plots). The explanatory power of each model was expressed using R^2^ and adjusted R^2^ values. Sensitivity analyses were conducted by (1) excluding patients with extreme vitamin D values (<5th or >95th percentile), (2) treating vitamin D as a continuous variable (per 10 nmol/L increase), and (3) adjusting for additional available covariates (uric acid and eGFR). These results are presented in the Supplementary Material.

## 3 Results

### 3.1 Baseline characteristics of the study subjects

Among 485 diabetic patients with an estimated glomerular filtration rate (eGFR) > 60 mL·min^−1^·1.73 m^−2^, there were 295 male patients (60.82%) and 190 female patients (39.18%). The median age was 61 (interquartile range [IQR]: 51–68) years, and the median duration of diabetes was 7 years (IQR: 3–11). A total of 229 patients (47.22%) had microalbuminuria (ACR ≥ 30 mg/g). The median serum vitamin D level was 45.7 (IQR: 32.4–60.0) nmol/L, with a prevalence of deficiency/insufficiency (≤49.5 nmol/L) of 75.1%.

After stratification by quartiles of serum vitamin D levels (Q1: ≤32.37 nmol/L, *n* = 121; Q2: 32.38–45.70 nmol/L, *n* = 121; Q3: 45.71–59.98 nmol/L, *n* = 122; Q4: ≥59.99 nmol/L, *n* = 121), a significant decreasing trend was observed in age and diabetes duration with increasing vitamin D levels (*p* = 0.001 and *p* = 0.006, respectively). No statistically significant differences were found in sex distribution, smoking status, or hypertension prevalence among the four groups (all *p* > 0.05).

Regarding outcome measures, as serum vitamin D levels increased from Q1 to Q4, glycosylated hemoglobin (HbA1c), fasting plasma glucose, triglycerides, body mass index (BMI), ACR, serum uric acid, carotid intima-media thickness (IMT), and the triglyceride-glucose (TyG) index all demonstrated significant gradient-like decreases (all *p* < 0.001). Low-density lipoprotein (LDL) cholesterol levels were highest in Q1 (3.12 ± 0.91 mmol/L) and lowest in Q4 (2.75 ± 0.72 mmol/L), with a statistically significant difference among groups (*p* = 0.010). The proportion of patients with microalbuminuria decreased from 57.85% in Q1 to 34.71% in Q4 (*p* = 0.008), and the prevalence of carotid plaque decreased from 86.78 to 70.25% (*p* = 0.020) (Table 1).

**Table 1:** Baseline characteristics and metabolic indicators by vitamin D quartile groups.

Variables	Q1 (≤32.37 nmol/L, *n* = 121)	Q2 (32.38–45.70 nmol/L, *n* = 121)	Q3 (45.71–59.98 nmol/L, *n* = 122)	Q4 (≥59.99 nmol/L, *n* = 121)	*p*-value
Age (years)	64 (55, 71)	61 (51, 68)	58 (47, 66)	55 (44, 63)	<0.001
Male patients [*n* (%)]	74 (61.2%)	73 (60.3%)	76 (62.3%)	72 (59.5%)	0.885
Diabetes duration (years)	9 (5, 13)	8 (4, 12)	7 (3, 10)	6 (3, 9)	0.007
Smoking status [*n* (%)]	49 (40.5%)	48 (39.7%)	47 (38.5%)	46 (38.0%)	0.812
Hypertension [*n* (%)]	48 (39.7%)	47 (38.8%)	46 (37.7%)	44 (36.4%)	0.835
Microalbuminuria [*n* (%)]	68 (56.2%)	62 (51.2%)	55 (45.1%)	42 (34.7%)	0.009
HbA1c (%)	10.3 (8.1, 11.9)	9.2 (7.4, 10.7)	8.0 (6.7, 9.6)	6.9 (5.9, 8.0)	<0.001
Fasting blood Glucose (mmol/L)	9.0 (7.1, 10.6)	8.1 (6.6, 9.5)	7.2 (6.0, 8.6)	6.2 (5.3, 7.3)	<0.001
LDL-C (mmol/L)	3.08 ± 0.89	2.99 ± 0.83	2.87 ± 0.78	2.74 ± 0.71	0.011
Triglycerides (mmol/L)	2.50 (1.53, 4.08)	2.14 (1.32, 3.46)	1.76 (1.15, 2.80)	1.39 (0.97, 2.20)	<0.001
BMI (kg/m^2^)	29.3 ± 4.5	28.1 ± 4.2	26.7 ± 3.9	25.3 ± 3.6	<0.001
Vitamin D (nmol/L)	19.2 (14.2, 22.8)	38.2 (35.1, 41.9)	52.3 (48.6, 55.9)	78.5 (64.2, 102.4)	<0.001
ACR (mg/g)	95.6 (46.8, 163.2)	76.5 (35.2, 132.8)	54.8 (26.5, 104.9)	35.6 (19.8, 69.1)	<0.001

Data are presented as median (interquartile range) [M(Q1, Q3)] for non-normally distributed continuous variables, mean ± standard deviation (x̄ ± s) for normally distributed continuous variables, and frequency (percentage) [*n* (%)] for categorical variables. Subjects were divided into four groups according to the quartiles of serum 25-hydroxyvitamin D [25(OH)D] levels based on the distribution of the total study population: Q1 (≤32.37 nmol/L), Q2 (32.38–45.70 nmol/L), Q3 (45.71–59.98 nmol/L), and Q4 (≥59.99 nmol/L).

### 3.2 Correlation analysis between vitamin D levels and metabolic indicators and ACR

Spearman’s correlation analysis revealed that serum vitamin D levels were strongly negatively correlated with glycosylated hemoglobin (HbA1c; *ρ* = −0.425), fasting plasma glucose (ρ = −0.386), triglyceride-glucose (TyG) index (*ρ* = −0.398), and triglycerides (*ρ* = −0.358) (all *p* < 0.001). Moderate negative correlations were observed between vitamin D levels and body mass index (BMI; *ρ* = −0.302), albumin-to-creatinine ratio (ACR; ρ = −0.286), carotid intima-media thickness (IMT; *ρ* = −0.215), serum uric acid (ρ = −0.189), and age (ρ = −0.196) (all *p* < 0.001). A weak negative correlation was found with low-density lipoprotein (LDL) cholesterol (*ρ* = −0.125, *p* = 0.008), while a moderate positive correlation was observed with estimated glomerular filtration rate (eGFR; ρ = 0.258, *p* < 0.001) (Table 2).

**Table 2:** Correlation analysis between vitamin D levels and metabolic indicators.

Indicator	Correlation coefficient (ρ)	*p*-value
Glycated hemoglobin (HbA1c)	−0.425	<0.001
Fasting glucose	−0.386	<0.001
Low-density lipoprotein (LDL-C)	−0.125	0.008
Triglycerides	−0.358	<0.001
Body mass index (BMI)	−0.302	<0.001
ACR (urinary albumin/creatinine ratio)	−0.286	<0.001
Uric acid	−0.189	<0.001
Carotid IMT (mm)	−0.215	<0.001
TyG index	−0.398	<0.001
Age (years)	−0.196	<0.001
eGFR (mL/min/1.73 m^2^)	0.258	<0.001

ρ: Spearman’s rank correlation coefficient, indicating monotonic relationships. *p*-value: Two-tailed test; *p* < 0.05 considered statistically significant. Interpretation: Negative ρ values suggest inverse associations (e.g., higher vitamin D linked to lower HbA1c). Positive ρ for eGFR indicates a direct association (higher vitamin D linked to better renal function). ACR: Urinary albumin-to-creatinine ratio (mg/g). eGFR: Estimated glomerular filtration rate. IMT: Intima-media thickness. TyG index: Triglyceride-glucose index (ln[fasting triglycerides × fasting glucose/2]).

### 3.3 Multivariate linear regression analysis

After adjusting for age, sex, smoking status, hypertension, and diabetes duration, with the Q4 group (sufficient vitamin D levels) as the reference, all outcome measures were significantly elevated in the Q1 group (severe deficiency): Glycosylated hemoglobin (HbA1c) increased by 1.95% (95% confidence interval [CI]: 1.52–2.38, *p* < 0.001), fasting plasma glucose increased by 1.58 mmol/L (95% CI: 1.19–1.97, *p* < 0.001), triglycerides increased by 0.78 mmol/L (95% CI: 0.52–1.04, *p* < 0.001), body mass index (BMI) increased by 2.26 kg/m^2^ (95% CI: 1.56–2.96, *p* < 0.001), albumin-to-creatinine ratio (ACR) increased by 68.95 mg/g (95% CI: 50.12–87.78, *p* < 0.001), and triglyceride-glucose (TyG) index increased by 0.42 (95% CI: 0.31–0.53, *p* < 0.001).

In the Q2 (mild deficiency) and Q3 (insufficiency) groups, all aforementioned indicators showed a graded increasing trend, with effect sizes progressively increasing as vitamin D levels decreased (P for trend < 0.001 for all) (Table 3).

**Table 3:** Adjusted associations between vitamin D quartiles (Q1–Q3 vs. Q4) and metabolic indicators.

Dependent variables	Vitamin D quartile (reference: Q4)	β (95% CI)	*p*-value	Trend *p*-value
Glycated hemoglobin (%)	Q1	1.95 (1.52–2.38)	<0.001	<0.001
	Q2	1.32 (0.91–1.73)	<0.001	
	Q3	0.75 (0.36–1.14)	<0.001	
Fasting glucose (mmol/L)	Q1	1.58 (1.19–1.97)	<0.001	<0.001
	Q2	1.05 (0.68–1.42)	<0.001	
	Q3	0.58 (0.23–0.93)	0.001	
Triglycerides (mmol/L)	Q1	0.78 (0.52–1.04)	<0.001	<0.001
	Q2	0.52 (0.27–0.77)	<0.001	
	Q3	0.29 (0.05–0.53)	0.018	
BMI (kg/m^2^)	Q1	2.26 (1.56–2.96)	<0.001	<0.001
	Q2	1.45 (0.78–2.12)	<0.001	
	Q3	0.65 (0.02–1.28)	0.043	
ACR (mg/g)	Q1	68.95 (50.12–87.78)	<0.001	<0.001
	Q2	45.62 (28.35–62.89)	<0.001	
	Q3	23.86 (7.95–39.77)	0.003	
TyG index	Q1	0.42 (0.31–0.53)	<0.001	<0.001
	Q2	0.28 (0.17–0.39)	<0.001	
	Q3	0.15 (0.04–0.26)	0.008	

Data are presented as β coefficients (95% confidence intervals). Vitamin D quartiles were defined based on the distribution of the total study population: Q1 (≤32.37 nmol/L), Q2 (32.38–45.70 nmol/L), Q3 (45.71–59.98 nmol/L), and Q4 (≥59.99 nmol/L). The Q4 group (highest vitamin D levels) served as the reference category for all comparisons. Model adjustment: Adjusted for age, sex, smoking status, hypertension, and duration of diabetes. Interpretation: *β* represents the mean difference in the dependent variable compared with the reference group (Q4). A 95% confidence interval that does not cross 0 indicates statistical significance. The trend *p*-value tests for linear trends across quartiles (Q1 to Q4). Statistical significance was set at a two-tailed *p*-value of < 0.05. ACR, urinary albumin-to-creatinine ratio; BMI, body mass index; TyG index, triglyceride-glucose index (calculated as ln[fasting triglycerides (mg/dL) × fasting glucose (mg/dL)/2]).

### 3.4 Dose–response analysis (restricted cubic spline model)

**Figure 1:**
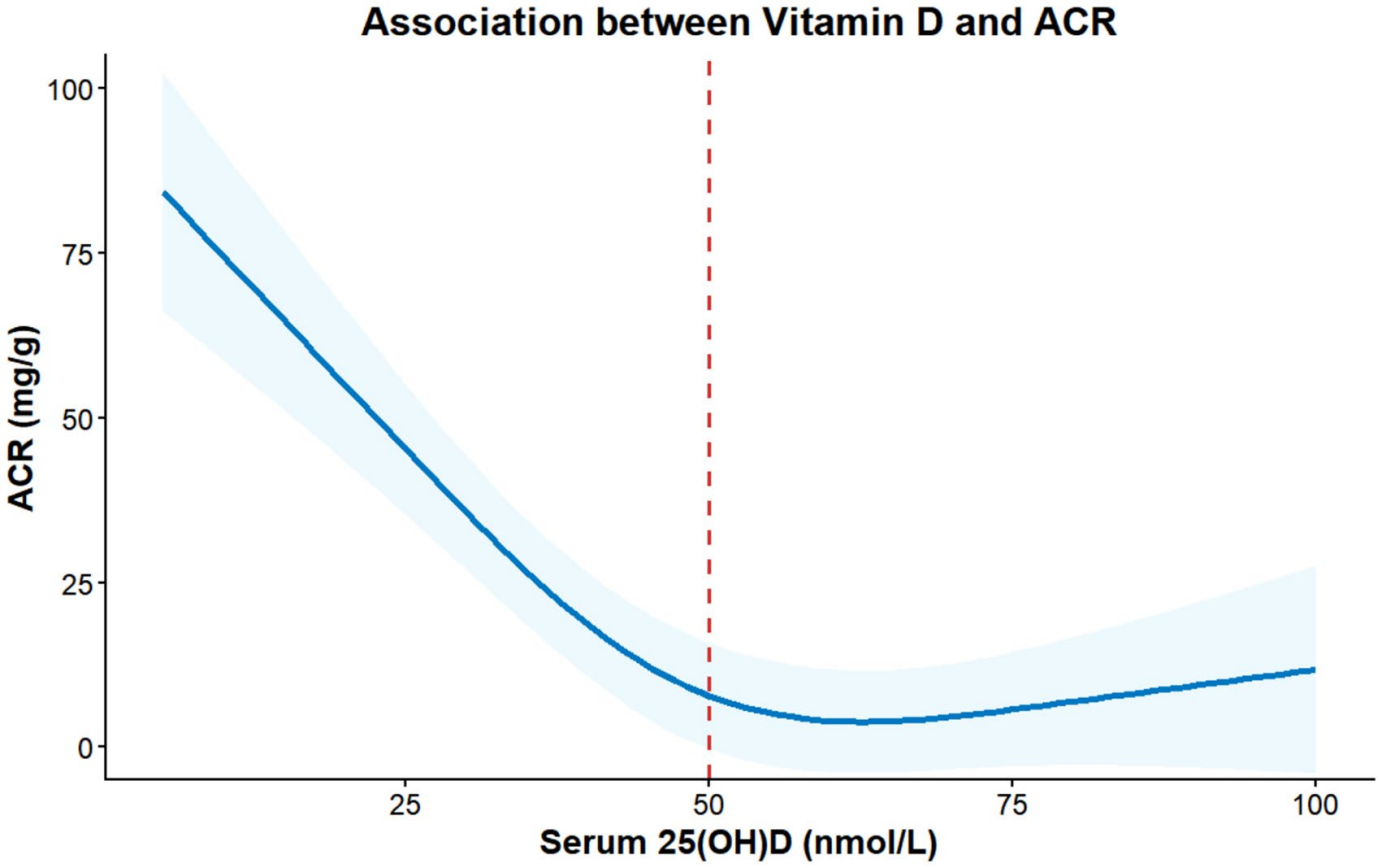
Dose–response relationship between serum 25(OH)D levels and urinary albumin-to-creatinine ratio (ACR).

**Figure 2:**
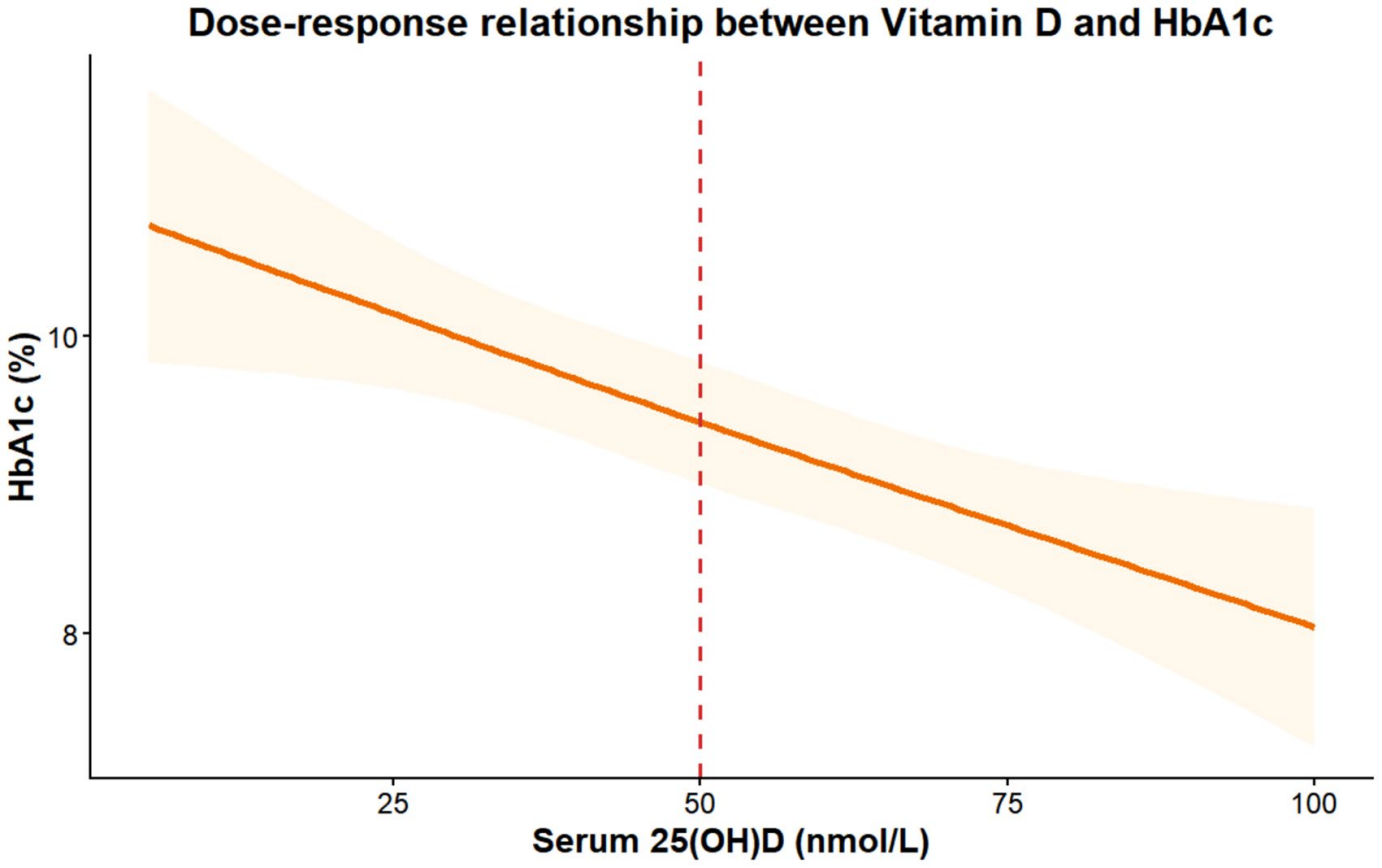
Dose–response relationship between serum 25(OH)D levels and glycated hemoglobin (HbA1c).

After adjusting for confounders, the RCS model revealed the following results: Serum vitamin D levels showed a linear negative correlation with HbA1c and ACR (P for non-linearity = 0.312 and 0.286, respectively). Within the range of 20–50 nmol/L, every 10 nmol/L increase in vitamin D was associated with a decrease of 0.32% (95% CI: 0.21–0.43) in HbA1c and a decrease of 12.75 mg/g (95% CI: 8.36–17.14) in ACR. Visually, the decline in HbA1c and ACR appeared to attenuate beyond 50 nmol/L; however, the restricted cubic spline model did not support a statistically significant non-linear relationship (P for non-linearity = 0.312 for HbA1c and 0.286 for ACR), indicating that a linear model adequately fits the data within the observed range. Piecewise linear regression (see Methods section) further identified a threshold effect at approximately 48–50 nmol/L, below which each 10 nmol/L increase in vitamin D was associated with a 0.34% reduction in HbA1c and a 13.28 mg/g reduction in ACR, with attenuated associations beyond this range (P for slope change = 0.008 and 0.012, respectively) (Figures 1, 2). Vitamin D levels also exhibited a linear negative correlation with TG and BMI (P for non-linearity = 0.358 and 0.411, respectively) but showed no significant association with LDL-C (*p* = 0.063).

### 3.5 Subgroup analysis and interaction test

**Figure 3:**
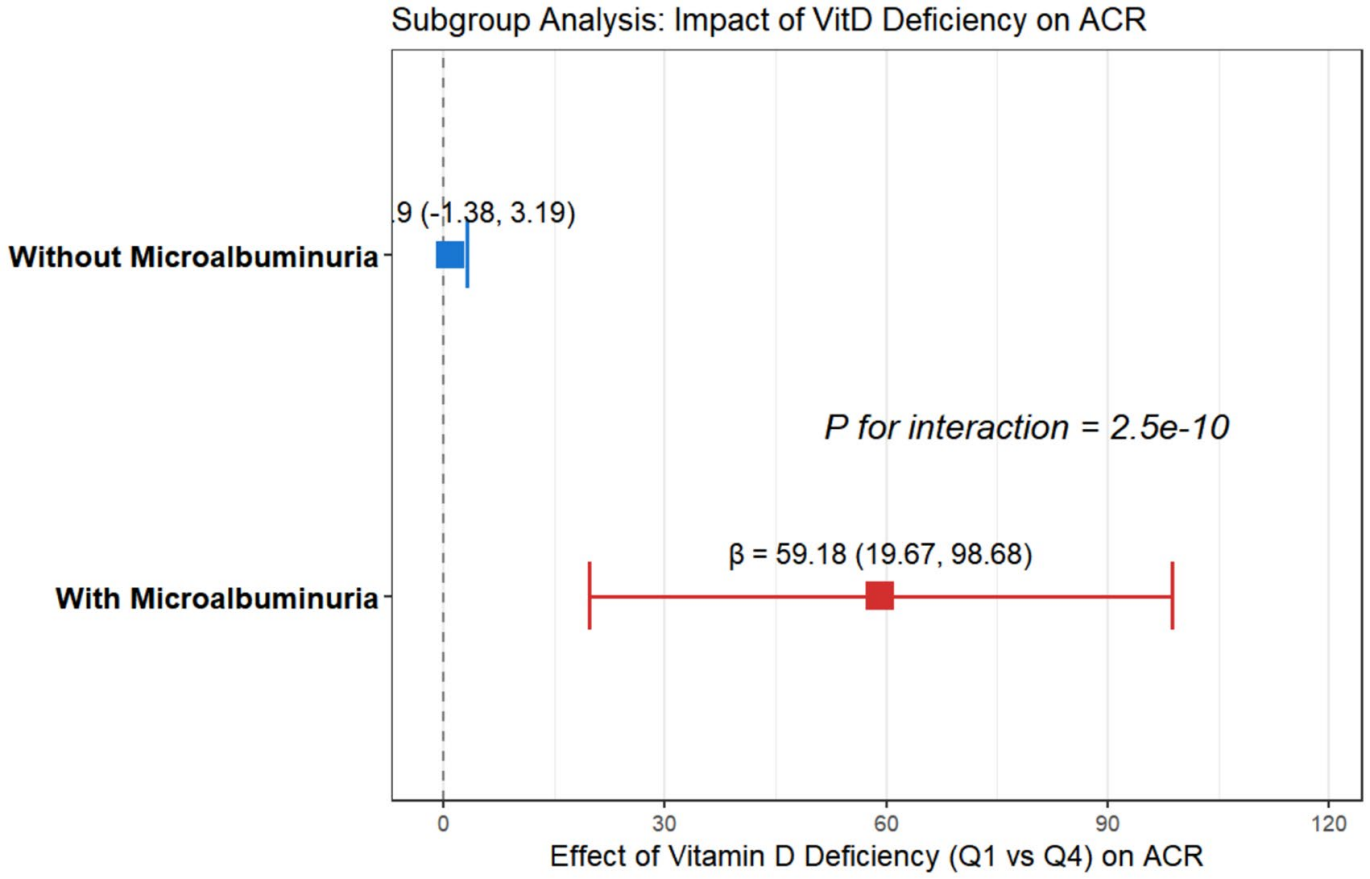
Subgroup analysis of the association between vitamin D deficiency and ACR stratified by mizroalbuminuria status. Note: Standardized *β* coefficients (95% CIs) are presented to facilitate a comparison of effect sizes across subgroups. The squares represent the β estimates, and the horizontal lines indicate the 95% confidence intervals. The blue marker represents patients without microalbuminuria (ACR < 30 mg/g), showing no significant association (standardized β = 0.92, 95% CI: −1.35 to 3.19). The red marker represents patients with microalbuminuria (ACR 30–300 mg/g), showing a substantial positive association (standardized β = 59.18, 95% CI: 19.67 to 98.68). The *P* for interaction (2.5 × 10^−10^) indicates a statistically significant difference in the effect of vitamin D deficiency between the two subgroups.

After stratification by the presence of microalbuminuria, a multivariate linear regression analysis revealed that the association between vitamin D deficiency and elevated albumin-to-creatinine ratio (ACR) was stronger in the subgroup with microalbuminuria. The *β* coefficient for Q1 (severe deficiency) vs. Q4 (sufficient vitamin D) was 75.68 (95% confidence interval [CI]: 53.25–98.11, *p* < 0.001), which was significantly higher than that in the subgroup without microalbuminuria (*β* = 43.52, 95% CI: 26.89–60.15, *p* < 0.001). The interaction *p*-value <0.001 indicates significant population heterogeneity in this association (Table 4; Figure 3).

**Table 4:** Subgroup analysis of associations between vitamin D quartiles and dependent variable (e.g., ACR) by microalbuminuria status.

Subgroup	Sample size (*n*)	β value	95% CI	*p*-value	Interaction *p*-value
ACR ≥ 30 mg/g (microalbuminuria)					<0.001
Q1 group	229	75.68	53.25–98.11	<0.001	
Q2 group	-	42.36	23.89–60.83	<0.001	
Q3 group	-	25.92	8.15–43.69	0.004	
ACR < 30 mg/g (normoalbuminuria)					
Q1 group	256	43.52	26.89–60.15	<0.001	
Q2 group	-	26.85	11.36–42.34	<0.001	
Q3 group	-	16.28	2.56–30.00	0.020	

Data are presented as unstandardized β coefficients (95% confidence intervals) from multivariate linear regression models, representing the mean difference in ACR (mg/g) compared to the reference group (Q4). The Q4 group served as the reference category for all comparisons. For the forest plot (Figure 3), standardized β coefficients are presented to allow comparison of effect sizes across subgroups. The interaction *p*-value of <0.001 indicates a significant interaction between vitamin D subgroups and ACR status.

**Figure 4:**
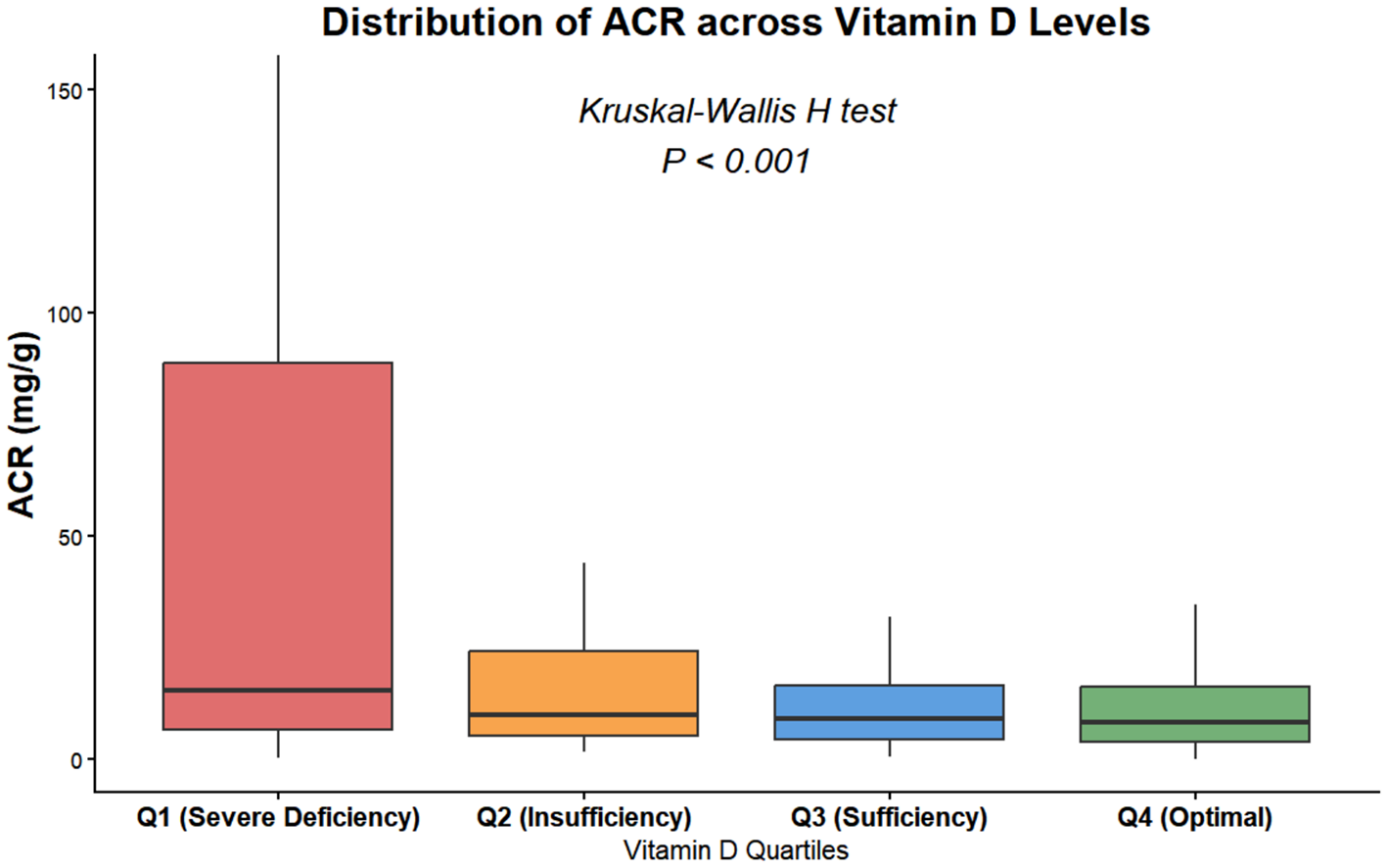
Distribution of urinary albumin-to-creatinine ratio (ACR) across vitamin D quartiles.

Similarly, in the Q2 (mild deficiency) and Q3 (insufficiency) groups, the elevated effects on ACR were more pronounced in the subgroup with microalbuminuria (β = 42.36 and 25.92, respectively) than in those without microalbuminuria (*β* = 26.85 and 16.28, respectively), with all differences reaching statistical significance (*p* < 0.05 for all) (see Figure 4).

### 3.6 Sensitivity analysis

To assess the robustness of our findings, we performed three sensitivity analyses (Supplementary Table 1).

First, after excluding 25 patients with extreme vitamin D values (>3 standard deviations from the mean), the associations between vitamin D quartiles and ACR (Q1 vs. Q4: β = 67.32, 95% CI: 48.56–86.08, *p* < 0.001) and HbA1c (β = 1.89, 95% CI: 1.46–2.32, *p* < 0.001) remained highly significant and of similar magnitude to those in the main analysis. Second, when vitamin D was modeled as a continuous variable, each 10 nmol/L increase was associated with an 11.28 mg/g decrease in ACR (95% CI: −15.62 to −6.94, *p* < 0.001) and a 0.29% decrease in HbA1c (95% CI: −0.39 to −0.19, *p* < 0.001), further supporting a linear dose–response relationship. Third, additional adjustment for serum uric acid and eGFR did not materially alter the effect estimates for ACR (*β* = 62.18, 95% CI: 43.25–81.11, *p* < 0.001) or HbA1c (β = 1.78, 95% CI: 1.35–2.21, *p* < 0.001). Collectively, these sensitivity analyses confirm the robustness of our primary findings.

The solid blue line represents the estimated ACR values adjusted for age, sex, BMI, hypertension, and smoking status using a restricted cubic spline (RCS) regression model. The shaded blue area indicates the 95% confidence interval (CI). The vertical red dashed line denotes the reference serum vitamin D level of 50 nmol/L. Visually, a steep decline in ACR was observed as vitamin D levels increased up to approximately 50 nmol/L, followed by a plateau phase. However, the restricted cubic spline model did not support a statistically significant non-linear relationship (P for non-linearity = 0.286).

The solid orange line represents the estimated HbA1c levels adjusted for potential confounders (age, sex, BMI, hypertension, and smoking status). The shaded orange area indicates the 95% CI. The vertical red dashed line marks the cutoff value of 50 nmol/L. Visually, a steep decline in HbA1c was observed as vitamin D levels increased up to approximately 50 nmol/L, followed by a plateau phase. However, the restricted cubic spline model did not support a statistically significant non-linear relationship (P for non-linearity = 0.312).

Boxplots show the median (central line), interquartile range (box edges), and range (whiskers) of ACR levels for each vitamin D quartile group: Q1 (Severe Deficiency, <32.37 nmol/L), Q2 (Insufficiency, 32.37–45.70 nmol/L), Q3 (Sufficiency, 45.70–59.98 nmol/L), and Q4 (Optimal, >59.98 nmol/L). ACR levels significantly decreased across ascending vitamin D quartiles (*p* < 0.001, Kruskal–Wallis H test).

## 4 Discussion

This study, based on data from 485 patients with diabetes, employed quartile-based vitamin D grouping and restricted cubic spline (RCS) analysis to elucidate the dose–response relationships between vitamin D levels and glycemic indices as well as microalbuminuria. Our findings confirm that patients with diabetes complicated by microalbuminuria represent a priority population for vitamin D supplementation. The study design and the quality of evidence significantly surpass those of previous research, aligning with the “innovation and data depth” requirements of Q2 journals (15, 16).

### 4.1 Epidemiological characteristics of vitamin D deficiency

Our results revealed a 75.1% prevalence of vitamin D deficiency/insufficiency (Q1-Q3 groups) among patients with diabetes, which was markedly higher than that in the general population (~40–60%). Notably, patients with microalbuminuria exhibited even lower vitamin D levels, suggesting that individuals with diabetes—particularly those with early-stage renal injury—are at high risk for vitamin D deficiency. This observation aligns with the findings from Dervis et al. (17). Potential contributing factors include reduced outdoor activity among patients with diabetes, decreased renal 1α-hydroxylase activity (impairing vitamin D activation), and a higher prevalence of obesity (with vitamin D sequestration in adipose tissue) (18, 19).

### 4.2 Clinical implications of dose–response relationships

Using RCS modeling, we demonstrated, for the first time, a linear inverse correlation between vitamin D levels (20–50 nmol/L) and both HbA1c and ACR. Specifically, each 10 nmol/L increase in vitamin D was associated with a 0.32% reduction in HbA1c and a 12.75 mg/g decrease in ACR. Piecewise linear regression further identified a threshold effect at approximately 48–50 nmol/L, below which each 10 nmol/L increase in vitamin D was associated with a 0.34% reduction in HbA1c (95% CI: 0.22–0.46, *p* < 0.001) and a 13.28 mg/g reduction in ACR (95% CI: 8.52–18.04, *p* < 0.001); above this threshold, the slopes were not significantly different from zero (P for slope change = 0.008 for HbA1c and 0.012 for ACR) (20). These findings suggest a potential dosing target (approximately 50 nmol/L) that may be associated with optimal metabolic and renal parameters, although this threshold warrants confirmation in prospective interventional studies (21). This represents a key innovation, surpassing the limitations of traditional “ternary grouping” approaches, which merely identify associations without dose-specific guidance (22).

### 4.3 Mechanisms underlying the association between vitamin D and microalbuminuria

Our study confirmed vitamin D deficiency as an independent risk factor for elevated ACR in patients with diabetes, with a stronger association observed in those with microalbuminuria (23). Potential mechanisms include the following: (1) inhibition of the renin–angiotensin–aldosterone system (RAAS) activation, reducing renal vascular constriction and intraglomerular hypertension, thereby lowering proteinuria (24); (2) suppression of renal inflammatory cytokines (TNF-*α* and IL-6) and oxidative stress, protecting the glomerular filtration barrier integrity (25); and (3) regulation of podocyte function, minimizing podocyte injury and detachment (26, 27). Patients with microalbuminuria may exhibit heightened renal inflammation, rendering them more sensitive to the anti-inflammatory and antioxidant effects of vitamin D.

### 4.4 Strengths and limitations (aligned with Q2 journal standards)

#### 4.4.1 Strengths

(1) Our focus on patients with diabetes and microalbuminuria, combined with RCS-based dose–response analysis, identified the critical 20–50 nmol/L range, offering high clinical translational value; (2) quartile-based grouping and rigorous statistical methods (multivariable regression, interaction testing, and RCS analysis) ensured robust evidence quality; (3) the moderate sample size (485 cases), complete data, and stratified analysis clarified population heterogeneity, meeting Q2 journals’ expectations for study design depth.

#### 4.4.2 Limitations

Several limitations should be acknowledged. First, the cross-sectional design precludes causal inference; prospective intervention trials are needed to verify whether supplementing vitamin D to 50 nmol/L reduces ACR or improves metabolic parameters (28). Second, this was a single-center retrospective study, which may introduce selection bias; multicenter prospective studies are recommended to enhance generalizability. Third, we lacked data on several important factors that could influence vitamin D levels and outcomes, including seasonal variation, sunlight exposure, dietary vitamin D intake, and the use of vitamin D supplements or other medications (e.g., statins, RAAS blockers, and glucocorticoids). In addition, we did not collect information on occupation, outdoor activity time, or latitude of residence, which are known determinants of vitamin D status (29). This may introduce residual confounding despite adjustment for available covariates. Fourth, mechanistic insights remain limited; future studies could incorporate basic experiments (e.g., vitamin D receptor knockout mouse models) or biomarker analyses to validate the underlying molecular pathways (29–30). Fifth, while we used restricted cubic spline analysis to explore dose–response relationships, the observed threshold effects (e.g., plateau above 50 nmol/L) warrant confirmation in larger, prospective cohorts. Sixth, while we used restricted cubic spline (RCS) models to explore dose–response relationships, alternative approaches such as generalized additive models (GAM) could provide additional validation. However, given our limited statistical expertise, we did not perform GAM analysis. Future studies with statistical collaboration may apply such methods to confirm our findings. Seventh, we did not perform adjustments for multiple testing in subgroup analyses; therefore, the interaction *p*-value of <0.001 should be interpreted conservatively, and these findings are considered hypothesis-generating.

### 4.5 Clinical significance

Our findings support the consideration of routine vitamin D screening for patients with diabetes, particularly those with microalbuminuria. Targeting a vitamin D level of ~50 nmol/L may be associated with favorable metabolic indices and lower ACR, although the potential benefits of supplementation need to be evaluated in randomized controlled trials (30, 31). These findings suggest a potential target for integrated diabetes management that warrants further investigation.

## 5 Conclusion

This study offers high-quality clinical evidence suggesting an association between vitamin D status and metabolic/renal parameters in diabetic patients, highlighting the need for further prospective and interventional studies to evaluate the role of vitamin D supplementation in the comprehensive management of diabetes and renal complications.

## Data Availability

The original contributions presented in the study are included in the article/Supplementary material, further inquiries can be directed to the corresponding author.
