# Supplementary figures and images for: Serum vitamin D levels correlate with metabolic abnormalities and microalbuminuria in diabetic patients: a restricted cubic spline dose–response analysis
Source: Front Nutr. 2026 May 19;13:1811665. doi: 10.3389/fnut.2026.1811665 (PMC13229412; doi:10.3389/fnut.2026.1811665)

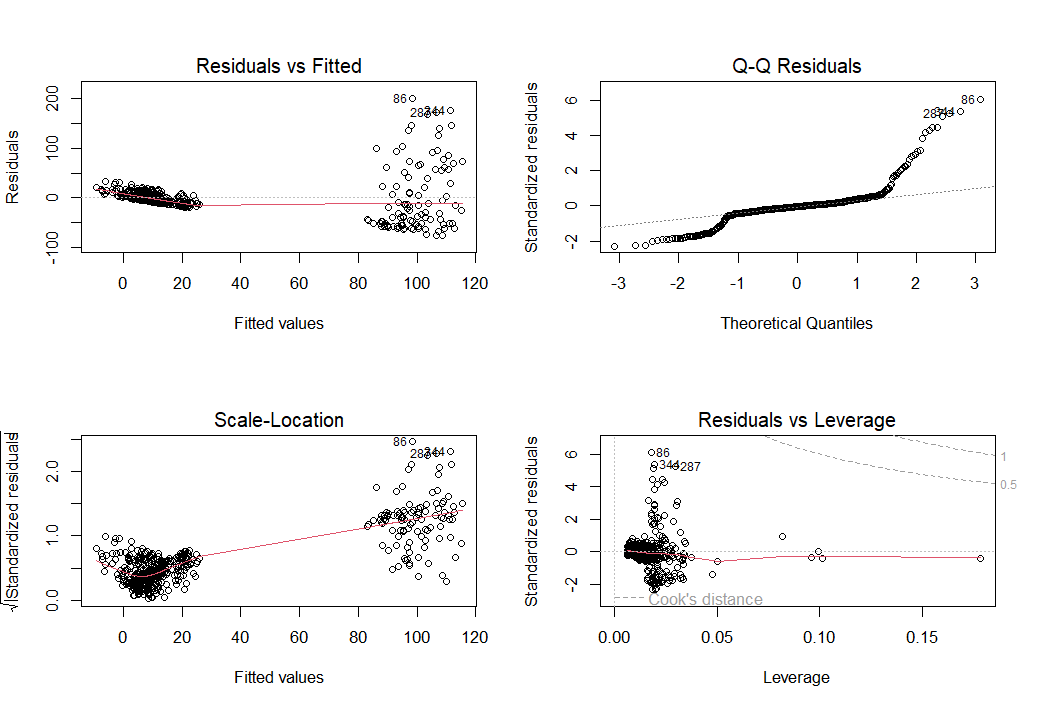

Supplement: Supplementary file 1 [file Image_1.TIFF]
