# Supplementary material for: Serum vitamin D levels correlate with metabolic abnormalities and microalbuminuria in diabetic patients: a restricted cubic spline dose–response analysis
Source: Front Nutr. 2026 May 19;13:1811665. doi: 10.3389/fnut.2026.1811665 (PMC13229412; doi:10.3389/fnut.2026.1811665)
Supplement: Supplementary file 2 [file Table_1.docx]

### Supplementary Table 1. Sensitivity analyses for the association between vitamin D and ACR/HbA1c

| Sensitivity analysis | Outcome | Comparison / Unit | β (95% CI) | P-value | Consistency with main analysis |
| --- | --- | --- | --- | --- | --- |
| **Exclusion of vitamin D outliers** (n=460) | ACR (mg/g) | Q1 vs Q4 | 67.32 (48.56–86.08) | <0.001 | ✓ Consistent |
|  | HbA1c (%) | Q1 vs Q4 | 1.89 (1.46–2.32) | <0.001 | ✓ Consistent |
| **Vitamin D as continuous variable** (n=485) | ACR (mg/g) | Per 10 nmol/L increase | -11.28 (-15.62 to -6.94) | <0.001 | ✓ Consistent |
|  | HbA1c (%) | Per 10 nmol/L increase | -0.29 (-0.39 to -0.19) | <0.001 | ✓ Consistent |
| **Additional adjustment for uric acid and eGFR** (n=485) | ACR (mg/g) | Q1 vs Q4 | 62.18 (43.25–81.11) | <0.001 | ✓ Consistent |
|  | HbA1c (%) | Q1 vs Q4 | 1.78 (1.35–2.21) | <0.001 | ✓ Consistent |

**Notes to Supplementary Table 1:**

Outliers were defined as vitamin D values >3 standard deviations from the mean (n=25 excluded).

All models were adjusted for age, sex, smoking status, hypertension, and diabetes duration.

For the continuous vitamin D analysis, the β represents the change in outcome per 10 nmol/L increase in serum vitamin D.

Abbreviations: ACR, albumin-to-creatinine ratio; eGFR, estimated glomerular filtration rate; HbA1c, glycated hemoglobin; Q, quartile.
